# Physicians’ views of performance reports: grading the graders

**DOI:** 10.1186/2045-4015-1-27

**Published:** 2012-06-20

**Authors:** Bruce E Landon

**Affiliations:** 1 Department of Health Care Policy, Harvard Medical School, 180 Longwood Avenue, Boston, MA, 02115, USA; 2 Division of General Medicine and Primary Care, Department of Medicine, Beth Israel Deaconess Medical Center, Boston, MA, USA

## Abstract

Quality measurement and feedback programs have become widespread and are looked upon as a cornerstone of quality improvement efforts. Often, such programs are used to motivate consumer choice through public report cards or to reward high quality care through pay for performance programs. Physicians’ views on performance measurement and feedback programs, however, are rarely sought, despite the potential usefulness for improving the impact of such programs. The new IJHPR paper by Nissanholtz-Gannot and colleagues provides important data on physicians’ views of the Israeli quality measurement program that demonstrates strong support for the program among Israeli physicians while also identifying potential areas for improvement.

## 

Over the past two decades, quality measurement and performance feedback programs have become widespread in the United States. Beginning with the a small number of process measures defined by the National Committee for Quality Assurance, the Healthcare Effectiveness Data and Information Set (HEDIS) program has grown to encompass dozens of measures across multiple domains of care and is widely reported by health plans and others throughout the United States. Beginning in 2000, Israel implemented a similar quality monitoring program for its health plans (the National Quality Monitoring Program, NQMP) that was based on the US HEDIS program that has been widely accepted by health plan managers and policy makers and credited with leading to important improvements in measured quality [1].

It is clear that the widespread adoption of quality measurement and feedback programs, both internal and external to physician practices will only continue to accelerate. It is widely acknowledged that quality of care across health systems is suboptimal and that systematic approaches to care based on a foundation of scientific measurement and management approaches will be crucial as our systems evolve from relying on the primacy of the individual physician to one based on systems and teams of care that come together to optimize the care delivered to our patients [2].

In both the US and Israel, however, anecdotal reports suggest that physicians are not fully supportive of these programs. Reasons cited include lack of agreement with the measures, concerns that such programs focus attention on aspects of care that can be measured at the expense of aspects of care that cannot be measured, concerns about unintended consequences (e.g., exasperating disparities in care), and insufficient adjustments for factors thought largely to be beyond the control of individual physicians such as the case mix of their population or patient adherence to recommendations [3]. There also are concerns that such systems lead to improvements in documentation, rather than fundamental improvements in quality of care. Finally, many physicians believe that such systems may be an affront to their notions of autonomy.

To the extent that such views are widespread among physicians, they might undermine the effectiveness of performance measurement and feedback systems. Thus, it becomes important to know more about physicians’ views of such programs. Thus, the paper is this issue by Nisshanholtz-Gannot and colleagues [4] is welcome and provides much needed information on how physicians view these programs, and the first such data in over a decade. Although the study is limited to Israeli physicians, it remains among the best to date and likely will be applicable to other systems of care, and, in particular, the US, whose quality monitoring system most closely resembles that of Israel. The authors surveyed over 600 primary care physicians (PCPs) working with all four Israeli health plans, and achieved a response rate of 69% among eligible physicians. The survey covered a broad array of topics related to the quality measurement program.

The Israeli quality monitoring system currently tracks 40 or so indicators in the areas of preventive services and care of patients with chronic conditions such as diabetes and asthma. Of note, and in contrast to most quality measurement programs in the US, neither plans nor physicians are rewarded for high performance and plan specific data were only recently released, and this was done in a manner that makes plan-level comparisons difficult. Thus, the primary focus of the program is on motivating internal quality improvement (although it is certainly quite likely that the specter of more transparent public reporting and pay for performance hang like a sword of Damocles ready to be dropped if quality is thought to be insufficient or not improving in an expected trajectory).

In general, the survey results demonstrate broad support for the quality measurement program among Israeli PCPs. Almost all physicians felt the program was important or very important and supported the continuation of the program. Even more importantly, half of physicians had made changes in their practices to improve performance. Only a small minority of physicians (9%) felt that the program led to deterioration of their relationships with patients, whereas over half felt that their relationships had improved. In general, the broad support among physicians allays concerns that physicians will work to undermine the effectiveness of the program.

There were, however, some reasons for concern as well as opportunities to improve the program. Although support was broad, about one-third of physicians felt that monitoring led to unnecessary treatment or tests and two-thirds felt that their workload had increased amid excessive management pressure to enhance performance on the measures. Similarly, about two-thirds of physicians felt that adherence to the standards distracted them from other clinical issues to a moderate or great extent and 20% felt that the program had impacted their job satisfaction negatively. Finally, about one-third of respondents felt that some of the indicators needed to be modified and that measurement programs need to take into account other factors that might contribute to care including general health, psychosocial, and sociodemographic status. This is of particular importance given that the Israeli program is simply informational, without the “teeth” of many programs that have been instituted elsewhere such as the UK or US. It is likely that physicians subject to more transparent public reporting or pay for performance based on these reports would feel more strongly about these issues.

Finally, it is important to develop deeper understanding of the processes by which *measurement* of quality is translated into *improvements* in quality. Given the broad adoption of electronic health records in Israel and the availability of data from these records, the Israeli quality-monitoring program is based on routine clinical data that is available for all applicable members. Despite this uniform substrate, it is likely that there are myriad approaches taken to improving performance on these measures. These may range from the use of population health managers and outreach personnel to the incorporation of electronic tools such as prompts or reminders or the empowerment of clinical team members to implement recommended screening and monitoring by protocol. The approaches used will be specific to the context of the practice related to both the practice organization and structure and the patient population served, but it is likely that effective approaches can be disseminated. Thus, although the current study provides data on the acceptance of the Israeli NQMP, it does little to add to our understanding of the effectiveness of various approaches taken towards improving performance on these measures. A previous study by the authors relates to this issue, although its focus is at the level of the health plan, and not approaches taken by physician practices within the plan [5].

There also are many questions left unanswered by this study and others that are of importance to the field. The NQMP focuses on a somewhat smaller set of measures than does HEDIS and far fewer than the Quality and Outcomes Framework implemented in the United Kingdom. What is the optimal number of measures to track? Does the inclusion of more measures lead to increased adoption of across the board quality improvement programs or, alternatively, does this increase the likelihood of unintended problems with unmeasured aspects of care? How can the design of measures be improved, particular given the availability of electronic clinical data in Israel? For instance, rather than simply focusing on control of LDL, the appropriateness of treatment can also be examined. For instance, perhaps credit should be given for appropriate treatment (e.g., maximal dose of a statin), even if the goal of LDL cholesterol level is not achieved. Thus, the availability of clinical data in the Israeli quality monitoring system, in contrast to the situation in the U.S., creates opportunities for the development of more clinically nuanced measures that might be even better accepted by physicians while also decreasing excessive attention given to meeting measures that are not always clinically appropriate.

Understanding the views of physicians is important to gauging the impact of performance improvement programs. Just as these programs seek to continuously improve the care delivered to patients, quality measurement programs themselves must be subjected to ongoing monitoring to improve the quality and usability of the program for those on the front lines delivering care. Thus, the data presented by studies such as the one reported in this issue will be of continuing importance as we strive to implement and improve upon programs designed to improve the quality and reliability of care.

## Author information

Bruce E. Landon, M.D., M.B.A., M.Sc. is Professor of Health Care Policy and Medicine at Harvard Medical School. He practices internal medicine at the Beth Israel Deaconess Medical Center. Dr. Landon's primary research interest has been assessing the impact of different characteristics of physicians and health care organizations, ranging from health plans to physician group practices, on the provision of health care services.

## Funding/support

None

## Financial disclosures

No relevant disclosures.
